# Supplementary material for: Predictors of Psychological Well-Being and Quality of Life in Patients with Hypertension: A Longitudinal Study
Source: Healthcare (Basel). 2024 Mar 9;12(6):621. doi: 10.3390/healthcare12060621 (PMC10969819; doi:10.3390/healthcare12060621)

HLM Model

Level-1 Model

$DV_{ti} = \pi_{0i} + \pi_{1i}*(Time_{ti}) + \pi_{2i}*(SBP_{ti}) + \pi_{3i}*(DBP_{ti}) + \pi_{4i}*(Composite.Risk.Factor_{ti}) +$
$\pi_{5i}*(LoC.Internal_{ti}) + \pi_{6i}*(LoC.External_{ti}) + \pi_{7i}*(RSES_{ti}) + e_{ti}$

Level-2 Model

$\pi_{0i} = \beta_{00} + \beta_{01}*(Randomization.Group_i) + \beta_{02}*(Sex_i) + \beta_{03}*(Previous.CVD_i) +$
$\beta_{04}*(End.Organ.Damage_i) + \beta_{05}*(Number.of.AHP.drugs.taken_i) + \beta_{06}*(STAI.Y_i) +$
$\beta_{07}*(DS\text{-}14_i) + \beta_{08}*(TYPE.A_i) + r_{0i}$

$\pi_{1i} = \beta_{00} + \beta_{11}*(Randomization.Group_i) + \beta_{12}*(Sex_i) + \beta_{13}*(Previous.CVD_i) +$
$\beta_{14}*(End.Organ.Damage_i) + \beta_{15}*(Number.of.AHP.drugs.taken_i) + \beta_{16}*(STAI.Y_i) +$
$\beta_{17}*(DS\text{-}14_i) + \beta_{18}*(TYPE.A_i) + r_{1i}$

$\pi_{2i} = \beta_{20}$

$\pi_{3i} = \beta_{30}$

$\pi_{4i} = \beta_{40}$

$\pi_{5i} = \beta_{50}$

$\pi_{6i} = \beta_{60}$

$\pi_{7i} = \beta_{70}$

Note. DV = Dependent Variable; CVD = Cardiovascular Disease; AHP = Anti-Hypertensive; STAY-Y = State Trait Anxiety Inventory, Trait subscale; DS-14 = Type D Scale 14; SBP = Systolic Blood Pressure; DBP = Diastolic Blood Pressure; LoC = Locus of Control; RSES = Rosenberg Self-Esteem Scale.

Supplementary Table S1.

Fixed effects of all predictors on **PGWB scores** at baseline (Intercept.2) and on its longitudinal changes over time ("Time" slope). The table further report the effects of the Time Varying Covariates (e.g., LoC and RSES) on the dependent variable.

Fixed Effect	Coefficient	SE	*t*-ratio	*df*	*p*-value
For INTERCEPT.1, π_0					
INTERCEPT.2, β_{00}	78.715935	1.044419	75.368	146	<0.001
Randomization Group, β_{01}	1.813319	1.997589	0.908	146	0.366
Sex, β_{02}	0.999276	2.011894	0.497	146	0.620
Previous CVD, β_{03}	3.535707	4.633663	0.763	146	0.447
End Organ Damage, β_{04}	0.378548	0.825420	0.459	146	0.647
Number of AHP drugs taken, β_{05}	-13.331651	8.697439	-1.533	146	0.127
**STAI-Y Trait, β_{06}**	-1.141051	0.168836	**-6.758**	146	**<0.001**
DS-14, β_{07}	0.201411	0.134753	1.495	146	0.137
Type A, β_{08}	-0.820250	0.430700	-1.904	146	0.059
For "Time" slope, π_1					
INTERCEPT.2, β_{10}	-0.027121	0.027877	-0.973	146	0.332
Randomization Group, β_{11}	0.003979	0.048915	0.081	146	0.935
Sex, β_{12}	0.007735	0.049546	0.156	146	0.876
Previous CVD, β_{13}	-0.143506	0.145690	-0.985	146	0.326
End Organ Damage, β_{14}	0.035791	0.017731	2.018	146	0.045
Number of AHP drugs taken, β_{15}	-0.052499	0.198078	-0.265	146	0.791
**STAI-Y Trait, β_{16}**	0.007714	0.003876	**1.990**	146	**0.048**
DS-14, β_{17}	-0.004964	0.003443	-1.442	146	0.152

**Type A, β_{18}**	0.034754	0.010542	**3.29**7	146	**0.001**
For "Clinical SBP" slope, π_2					
INTERCEPT.2, β_{20}	-0.111877	0.055006	-2.034	115	0.044
For "Clinical DBP" slope, π_3					
INTERCEPT.2, β_{30}	-0.165499	0.079505	-2.082	115	0.040
For "Composite Risk Factor" slope, π_4					
INTERCEPT.2, β_{40}	0.046301	1.039928	0.045	115	0.965
For "**Internal LoC**" slope, π_5					
INTERCEPT.2, β_{50}	0.343459	0.133464	**2.57**3	115	**0.011**
For "**External LoC**" slope, π_6					
INTERCEPT.2, β_{60}	-0.255204	0.082676	**-3.087**	115	**0.003**
For "**RSES**" slope, π_7					
INTERCEPT.2, β_{70}	1.083358	0.167101	**6.483**	115	**<0.001**

Supplementary Table S2.

Fixed effects of all predictors on **SF-36 PCS** at baseline (Intercept.2) and on its longitudinal changes over time (“Time” slope). The table further report the effects of the Time Varying Covariates (e.g., LoC and RSES) on the dependent variable.

Fixed Effect	Coefficient	SE	*t*-ratio	*df*	*p*-value
For INTERCEPT.1, π_0					
INTERCEPT.2, β_{00}	49.210908	0.595315	82.664	146	<0.001
Randomization Group, β_{01}	-0.258758	1.268524	-0.204	146	0.839
Sex, β_{02}	4.277522	1.413320	3.027	146	0.003
Previous CVD, β_{03}	-2.496148	3.068092	-0.814	146	0.417
End Organ Damage, β_{04}	1.110865	0.507715	2.188	146	0.030
Number of AHP drugs taken, β_{05}	-15.227654	4.528751	-3.362	146	<0.001
STAI-Y Trait, β_{06}	-0.155319	0.088975	-1.746	146	0.083
DS-14, β_{07}	-0.024309	0.082476	-0.295	146	0.769
Type A, β_{08}	-0.411583	0.360240	-1.143	146	0.255
For “Time” slope, π_1					
INTERCEPT.2, β_{10}	-0.020728	0.016416	-1.263	146	0.209
Randomization Group, β_{11}	0.031297	0.032304	0.969	146	0.334
Sex, β_{12}	-0.013578	0.036371	-0.373	146	0.709
Previous CVD, β_{13}	-0.050682	0.073246	-0.692	146	0.490
End Organ Damage, β_{14}	-0.023079	0.011941	-1.933	146	0.055
Number of AHP drugs taken, β_{15}	0.191394	0.115431	1.658	146	0.099
**STAI-Y Trait, β_{16}**	-0.005535	0.002360	**-2.345**	146	**0.020**
DS-14, β_{17}	0.002398	0.002083	1.151	146	0.252
Type A, β_{18}	0.008773	0.008716	1.007	146	0.316
For “Clinical SBP” slope, π_2					
INTERCEPT.2, β_{20}	0.012636	0.034896	0.362	111	0.718
For “Clinical DBP” slope, π_3					
INTERCEPT.2, β_{30}	-0.025529	0.048569	-0.526	111	0.600
For “Composite Risk Factor” slope, π_4					
INTERCEPT.2, β_{40}	0.899202	0.555263	1.619	111	0.108
For “**Internal LoC”** slope, π_5					
INTERCEPT.2, β_{50}	0.275086	0.109723	**2.507**	111	**0.014**
For “External LoC” slope, π_6					
INTERCEPT.2, β_{60}	-0.114461	0.070493	-1.624	111	0.107

For “RSES” slope, π_7					
INTERCEPT.2, β_{70}	0.224777	0.141846	1.585	111	0.116

Supplementary Table S3.

Fixed effects of all predictors on **SF-36 MCS** at baseline (Intercept.2) and on its longitudinal changes over time ("Time" slope). The table further report the effects of the Time Varying Covariates (e.g., LoC and RSES) on the dependent variable.

Fixed Effect	Coefficient	SE	*t*-ratio	*df*	*p*-value
For INTERCEPT.1, π_0					
INTERCEPT.2, β_{00}	45.648242	0.751742	60.723	146	<0.001
Randomization Group, β_{01}	-0.677145	1.555237	-0.435	146	0.664
Sex, β_{02}	-0.528593	1.405244	-0.376	146	0.707
Previous CVD, β_{03}	2.958204	2.816695	1.050	146	0.295
End Organ Damage, β_{04}	-0.031428	0.561186	-0.056	146	0.955
Number of AHP drugs taken, β_{05}	-4.616898	5.037851	-0.916	146	0.361
**STAI-Y Trait, β_{06}**	-0.494523	0.109127	**-4.532**	146	**<0.001**
DS-14, β_{07}	0.010591	0.093181	0.114	146	0.910
Type A, β_{08}	-0.460294	0.340527	-1.352	146	0.179
For "Time" slope, π_1					
INTERCEPT.2, β_{10}	0.019687	0.020974	0.939	146	0.349
Randomization Group, β_{11}	0.044133	0.041963	1.052	146	0.295
Sex, β_{12}	0.053351	0.038732	1.377	146	0.170
Previous CVD, β_{13}	-0.083486	0.058486	-1.427	146	0.156
End Organ Damage, β_{14}	0.036291	0.011578	3.135	146	0.002
Number of AHP drugs taken, β_{15}	-0.156674	0.117773	-1.330	146	0.185
**STAI-Y Trait, β_{16}**	0.005570	0.002772	**2.009**	146	**0.046**
DS-14, β_{17}	-0.002459	0.002435	-1.010	146	0.314
**Type A, β_{18}**	0.020484	0.009026	**2.269**	146	**0.025**
For "Clinical SBP" slope, π_2					
INTERCEPT.2, β_{20}	-0.035852	0.046793	-0.766	111	0.445
For "Clinical DBP" slope, π_3					
INTERCEPT.2, β_{30}	-0.069892	0.059192	-1.181	111	0.240
For "Composite Risk Factor" slope, π_4					
INTERCEPT.2, β_{40}	-0.777615	0.908070	-0.856	111	0.394
For "Internal LoC" slope, π_5					
INTERCEPT.2, β_{50}	0.033244	0.113915	0.292	111	0.771
For "**External LoC**" slope, π_6					
INTERCEPT.2, β_{60}	-0.130371	0.065575	**-1.988**	111	**0.049**
For "**RSES**" slope, π_7					
INTERCEPT.2, β_{70}	0.630425	0.126559	**4.981**	111	**<0.001**

Note. SE = Standard Error; *df* = degrees of freedom; CVD = Cardiovascular Disease; AHP = Anti-Hypertensive; STAY-Y = State Trait Anxiety Inventory, Trait subscale; DS-14 = Type

D Scale 14; SBP = Systolic Blood Pressure; DBP = Diastolic Blood Pressure; LoC = Locus of Control; RSES = Rosenberg Self-Esteem Scale.

Supplementary Figure S1.

Significant effects of trait anxiety on psychological well-being (PGWB) from baseline up to 48-weeks of follow-up.

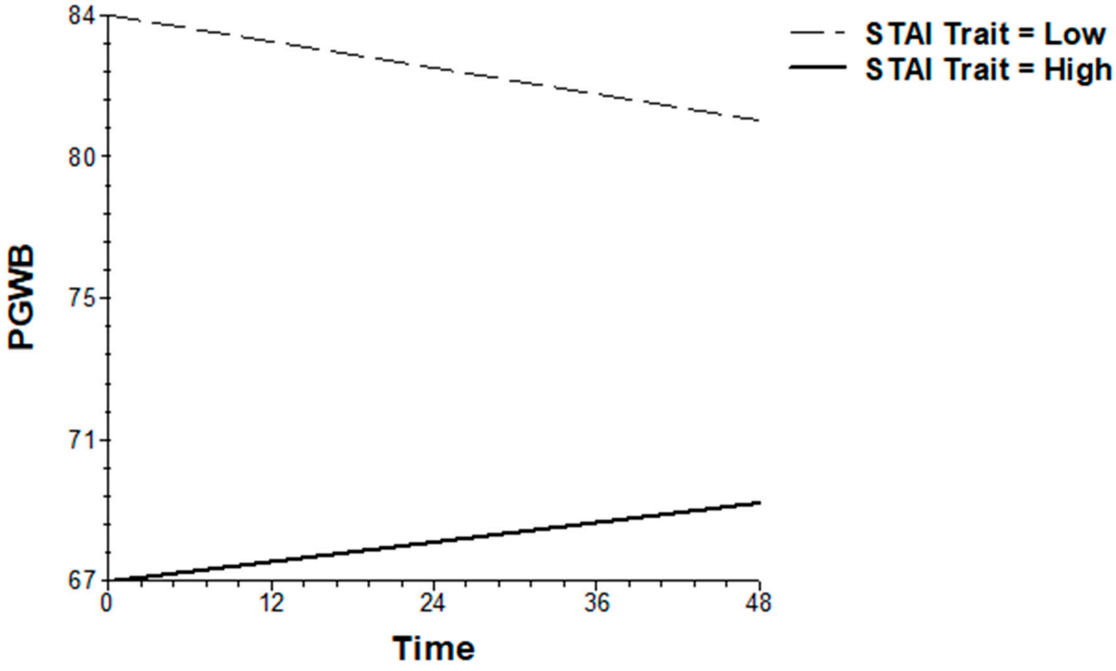

Supplementary Figure S2.

Significant effects of trait anxiety on health-related quality of life mental domain (SF-36 MCS) from baseline up to 48-weeks of follow-up.

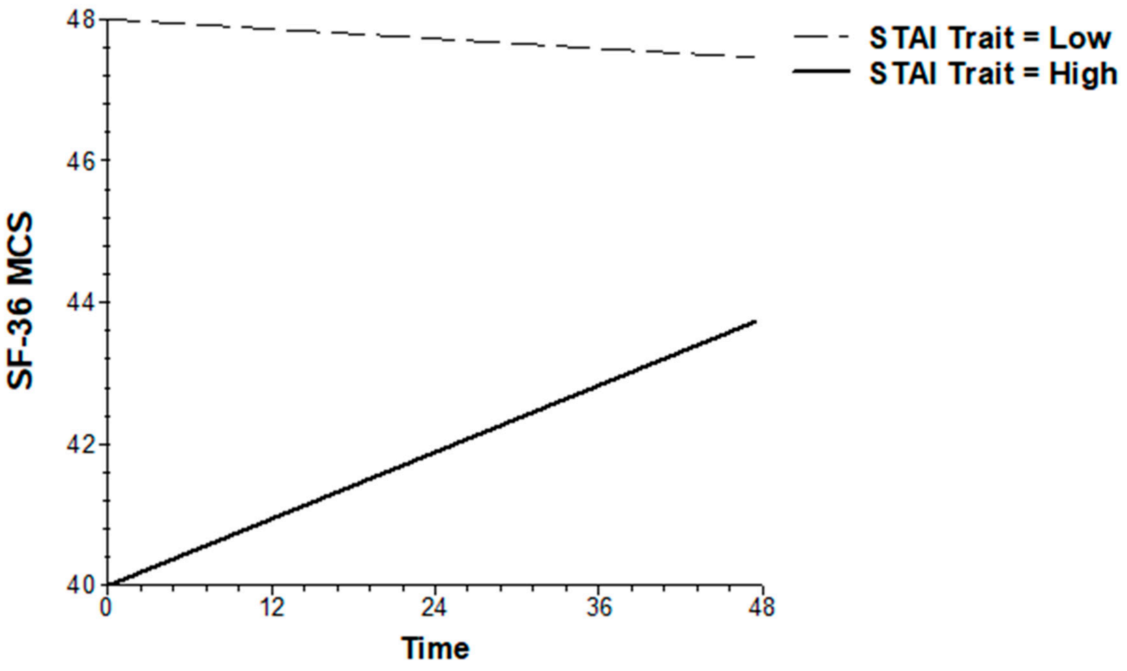

Supplement: Supplementary file 1 [file healthcare-12-00621-s001.zip › healthcare-2827009-supplementary.pdf]
